# Humulene Inhibits Acute Gastric Mucosal Injury by Enhancing Mucosal Integrity

**DOI:** 10.3390/antiox10050761

**Published:** 2021-05-11

**Authors:** Dahee Yeo, Su-Jung Hwang, Ye-Seul Song, Hyo-Jong Lee

**Affiliations:** 1 Institute of Pharmaceutical Sciences and Research, College of Pharmacy and Inje, Inje University, 607 Obang-dong, Gimhae 621749, Korea; me-ydh@hanmail.net (D.Y.); sama3575@naver.com (S.-J.H.); 2 School of Pharmacy, Sungkyunkwan University, 2066 Seobu-ro, Jangan-gu, Suwon 16419, Korea; summerai6533@naver.com

**Keywords:** α-humulene, gastritis, histamine, antioxidant, cytokine, mucins

## Abstract

This study was designed to determine whether α-humulene, a major constituent in many plants used in fragrances, has a protective role against gastric injury in vivo and in vitro. A rat model of hydrochloric acid (HCl)/ethanol-induced gastritis and human mast cells (HMC-1) were used to investigate the mucosal protective effect of α-humulene. α-Humulene significantly inhibited gastric lesions in HCl/ethanol-induced acute gastritis and decreased gastric acid secretion pyloric ligation-induced gastric ulcers in vivo. In addition, α-humulene reduced the amount of reactive oxygen species and malondialdehyde through upregulation of prostaglandin E2 (PGE2) and superoxide dismutase (SOD). In HMC-1 cells, α-humulene decreased intracellular calcium and increased intracellular cyclic adenosine monophosphate (cAMP) levels, resulting in low histamine levels. α-Humulene also reduced the expression levels of cytokine genes such as interleukin (IL)-1β, IL-6, tumor necrosis factor (TNF) by downregulating nuclear factor-κB (NF-κB) nuclear translocation. Finally, α-humulene upregulated the expression levels of mucin 5AC (Muc5ac), Muc6, trefoil factor 1 (Tff1), trefoil factor 2 (Tff2), and polymeric immunoglobulin receptor (pigr). α-Humulene may attenuate HCl/ethanol-induced gastritis by inhibiting histamine release and NF-κB activation and stimulating antioxidants and mucosal protective factors, particularly Muc5ac and Muc6. Therefore, these data suggest that α-humulene is a potential drug candidate for the treatment of stress-induced or alcoholic gastritis.

## 1. Introduction

Gastritis refers to the inflammation of the mucosal lining that protects the inner layers of the stomach. It can be classified as gastritis with damage only at the mucosal surface and gastric ulcer, with damage deeper than the mucosal layer. In general, gastritis is caused by alcohol, *Helicobacter pylori*, and some drugs and its major symptoms include loss of appetite, nausea, vomiting, and heartburn [1,2,3,4]. When alcohol is consumed, it directly reaches the mucus layer of the stomach and stimulates excessive gastric juice secretion [5]. Moreover, mast cells in the gastric tissue release histamine in large amounts [6], which consequently excites histamine receptors in parietal cells that produce acid [7]. As gastric acid secretion increases, damage to the mucus layer also increased. In addition, alcohol induces inflammatory activity, while decreasing antioxidant activity and mucus protective factors. These multiple factors work together, eventually leading to gastritis. Since the consumption of alcohol is still rising worldwide, many researchers have now focused on the treatment of alcoholic gastritis [8].

To develop novel anti-gastritic drugs, it is important to understand the underlying molecular mechanism of gastritis and its major targets. Among the many types of targets, the most important is histamine. Physical stress and inflammation induce the secretion of histamine and then excite histamine receptors on parietal cells, thus accelerating the production of gastric acid. Based on the importance of histamine action, histamine receptor blockers such as cimetidine and ranitidine are widely used to treat gastritis. However, cimetidine has some adverse effects because it targets not only histamine receptors but also androgen receptors [9]. Recently, it has been reported that ranitidine also has side effects such as anaphylactic shock. Therefore, there is a need to develop anti-gastritic medicines [10]. The second key target is oxidative stress, owing to generation of reactive oxygen species (ROS) [11]. When epithelial cells are injured, they utilize more oxygen and the amount of peroxide radicals rapidly increases [11]. Although these free radicals are promptly removed by several enzymatic reactions such as superoxide dismutase (SOD) and catalase, excessive oxidative stress can cause mucosal toxicity and damage the surrounding gastric tissues [11]. Finally, there are critical protective molecules in the gastric mucus, such as mucin 5AC (MUC5AC), mucin 6 (MUC6), trefoil factor 1 (TFF1), trefoil factor 2 (TFF2), and polymeric immunoglobulin receptor (pIgR) [12,13,14,15,16,17]. These molecules protect the gastric mucosa against offensive extrinsic factors such as alcohol and H. pylori. MUC5AC and MUC6 are secretory-type mucins and are highly expressed in the gastric mucosal epithelium to protect the gastric mucosa [13]. The TFF peptides (TFF1 and TFF2) are small secretory peptides with a 3-leaf structure; they are mainly expressed in the gastric mucosa, where they are involved in protection, stabilization, and healing of the mucosal layer [18,19]. Interestingly, TFF peptides are coexpressed with mucins in the gastrointestinal tract. For example, TFF1 appears with MUC5AC and TFF2 appears with MUC6 [14,20,21]. pIgR mediates transcytosis of polymeric immunoglobulins (pIgs), which are important for mucosal immunity [22]. For instance, pIgR directly interacts with mucosal IgM to protect the organism. Natural products and their constituents that decrease histamine and simultaneously increase mucosal protecting factors (mucin and TFFs) have sufficient potential and value to be developed as therapeutic agents for gastritis.

α-Humulene is also known as α-caryophyllene, a biogenic volatile monocyclic sesquiterpene (C_15_H_24_) [23], and its IUPAC name is (1*E*,4*E*,8*E*)-2,6,6,9-tetramethyl-1,4-8-cycloundeca-1,4,8-triene. It contains an eleven-membered ring with three *trans*-endocyclic C = C double bonds. In many aromatic plants, α-humulene is often present with its ring-opened isomer, β-caryophyllene, which has anti-cancer and anti-inflammatory activities [24,25]. Previously, it was reported that the essential oil containing α-caryophyllene exhibits anti-allergic activity on carrageenan-induced paw-edema in mice by reducing the level of tumor necrosis factor (TNF)-α [26]. Most natural products containing α-humulene have demonstrated anti-inflammatory effects, indicating that α-humulene is a potential anti-inflammatory agent [27,28]. However, it is largely unknown whether α-humulene has a protective role in HCl/ethanol-induced gastritis and how it affects the mucosal niche. This prompted us to study the anti-gastritis activity of α-humulene in vivo and *in vitro*. In this study, a HCl/ethanol-induced gastritis or pylorus ligation-induced ulcer rat model was utilized to reveal the protective effect of α-humulene and its related mechanism for mucus secretion, gastric acid secretion, oxidant/antioxidant balance, and mucosal stabilizing factors such as MUC5AC and MUC6 in vivo. In addition, we used the phorbol 12-myristate 13-acetate (PMA)-induced human mast cell-1 (HMC-1) model. PMA, a potent activator of protein kinase C, stimulates HMC-1 cells to exhibit characteristis of tissue mast cells including degranulation, surface antigen profile, and cytokine activation pathways. Thus, we examined the inhibitory effect of α-humulene and its underlying molecular mechanism of histamine release, oxidative stress, and NF-κB-mediated inflammatory responses in the HMC-1 cell line stimulated with PMA.

## 2. Materials and Methods

### 2.1. Reagents and Cell Culture

Human leukemic mast cell line (HMC-1) was purchased from the Korean Cell Line Bank (KCLB, Seoul, Korea) and cultured at 37 °C and 5% CO_2_ in Iscove’s modified Dulbecco’s medium supplemented with 10% fetal bovine serum, penicillin (100 U/mL), and streptomycin (100 μg/mL; Gibco BRL, Gaithersburg, MD, USA). α-Humulene, ranitidine, dimethyl sulfoxide (DMSO), Alcian Blue 8GX, curcumin, histamine, compound 48/80, phorbol-12-myristate-13-acetate (PMA), and o-phthalaldehyde were purchased from Sigma Chemicals (St. Louis, MO, USA). All antibodies were obtained from Cell Signaling Technology (Danvers, MA, USA).

### 2.2. Animals

Male Sprague-Dawley rats (200–250 g, 6 weeks old) were purchased from Orient Bio Inc. (Seoul, Korea) and housed under controlled conditions (temperature, 20–22 °C; humidity, 40–60%). All rats were fed standard laboratory chow and water ad libitum. The Institutional Animal Care and Use Committee (IACUC) of Inje University in Gimhae, South Korea (Inje-2015-21) approved the experiment procedures including handling and care of adult rats on 1 November 2015.

### 2.3. Induction of Gastric Injury in Rats by HCl/Ethanol Injection

The anti-gastritis activity of α-humulene was evaluated in rats with gastritis induced by 150 mM HCl/60% ethanol. The rats were randomly separated into six groups eight animal each and fasted for 24 h prior to the experiment but allowed to drink water until 4 h before the experiment. The control group received only vehicle (1.5 mL/200 g body weight), the positive control groups received ranitidine (10 mg/kg), and the experimental groups received α-humulene (20, 50, or 100 mg/kg) by oral gavage. After 30 min, all groups were orally administered 1.5 mL of 150 mM HCl/60% ethanol to induce gastric mucosal damage. After 1 h, the animals were euthanized. The stomachs were rapidly removed and fixed by inflation with 8 mL of 10% formalin. Then, the fixed stomachs were opened along the greater curvature and the amount of lesions on the inner face of the stomach was measured by summing the total length (mm) of each lesion using ImageJ (NIH) and expressed it in graph as a lesion index. In addition, gastric lesion inhibition (%) was calculated by the following formula:

gastric lesion inhibition (%) = [(length of negative control) − (length of drug administration)]/(length of negative control) × 100

### 2.4. Histological Examination and Gastric Wall Mucus Determination

For histological examination, the stomach tissues were sectioned to 4-μm thickness and then stained with hematoxylin and eosin. To estimate the mucus content, the amount of Alcian Blue bound to the gastric tissues was measured. Briefly, sectioned tissues were incubated with 3% acetic acid for 10 min, stained with 1% Alcian Blue 8GX, and then rinsed for 15 min under running tap water. The stain was eluted from the tissue with DMSO and the absorbance of each supernatant was measured at 650 nm. All images were obtained using an Axiovert M200 microscope (Zeiss, Oberkochen, Germany).

### 2.5. Gastric Anti-Secretory Activity

Anti-secretory activity of α-humulene was measure according to pylorus ligation techniques described by Shay and his colleagues [29]. Rats were randomly separated into three groups of eight animal each and starved for 24 h but had free access to water. Then, control group received only vehicle (1.5 mL/200 g body weight), the positive control groups received ranitidine (10 mg/kg), and the experimental groups received α-humulene (100 mg/kg) by oral gavage. After 1 h, the pylorus was ligated under anesthesia. Six hours later, the animals were euthanized under ether anesthesia. The gastric juice was collected from stomach and centrifuged at 3000 rpm for 15 min. The volume of the supernatant was measured and the acidity of gastric content was measured by titrating of the gastric juice with 0.1 N NaOH using phenol red as pH indicator.

### 2.6. Acid-Neutralizing Capacity

The acid-neutralizing capacity was measured by titration with 0.1 N NaOH using phenol red as pH indicator. Ranitidine and α-humulene were added to 100 mL of 0.1 N HCl (artificial gastric acid) and then incubated at 37 °C with shaking for 1 h. Calcium carbonate was used as a positive control.

### 2.7. MTS Cell Viability Assay

HMC-1 cells were exposed to various concentrations of α-humulene (62.5 to 1000 μM) in 96-well plates for 24 h. Twenty microliters of MTS reagent (3-(4,5-dimethylthiazol-2-yl)-5-(3-carboxymethoxyphenyl)-2-(4-sulfophenyl)-2H-tetrazolium, Promega, Madison, WI, USA) was added to each well and incubated for 4 h at 37 °C. The absorbance was then measured at 490 nm.

### 2.8. Histamine Assay

To evaluate the effect of α-humulene on the degranulation of HMC-1 cells, the level of histamine in the supernatant of the cultured cells was measured. In brief, HMC-1 suspensions in 500 µl of phosphate-buffered saline (PBS) were pre-incubated for 1 h with curcumin (50 µL/mL) as a positive control or α-humulene (250, 500 or 1000 μM). Subsequently, all groups were incubated with compound 48/80 (200 µg/mL) for 3 h, and then 24 µL of 1 N NaOH and 6 µL of *o*-phthalaldehyde (Sigma, St. Louis, MO, USA) were added to each well and incubated at room temperature for 4 min. Twelve microliters of 3 N HCl was added to terminate the reaction and the fluorescence intensity was measured with a Synergy™ HTX Multi-Mode Microplate Reader (BioTek Instruments, Winooski, VT, USA) using 360 nm excitation filter and 460 nm emission filter.

### 2.9. Measurement of Intracellular Calcium Level

To measure the concentrations of intracellular calcium in HMC-1 cells, Fura 2-acetoxymethyl ester (Fura/2AM, Invitrogen, Waltham, MA, USA), a membrane-permeant derivative of the ratiometric calcium indicator, was loaded into the cells for 1 h. After washing twice, the changes in intracellular calcium were detected using the Synergy™ HTX Multi-Mode Microplate Reader (BioTek Instruments).

### 2.10. Measurement of Intracellular Cyclic Adenosine Monophosphate (cAMP) Levels

The level of intracellular cAMP was measured by a cAMP enzyme immunoassay kit (GE Healthcare Life science, Buckinghamshire, UK) according to the manufacturer’s instructions. Briefly, HMC-1 cells (2 × 10^6^ cells/mL) were suspended in 500 µL of PBS and treated 1 mM of α-humulene for the indicated times. Then, ice-cold ethanol was added to stop the reaction and the mixture was centrifuged at 1200 rpm for 2 min. The sample was reconstituted in assay buffer and intracellular cAMP levels were determined by enzyme immunoassay.

### 2.11. Measurement of Prostagland E2 (PGE_2_) Levels

The gastric tissues were weighed and homogenized in the homogenization buffer (0.1 M phosphate (pH 7.4) buffer with 1 mM EDTA). The homogenates were centrifuged at 13,000 rpm for 15 min at 4 °C, the supernatant was collected and PGE_2_ levels were determined by PGE_2_ ELISA kit (Cayman Chemical Co., Ann Arbor, MI, USA) according to the manufacturer’s instructions.

### 2.12. Measurement of Malondialdehyde (MDA) Levels

MDA level was determined by a thiobarbituric acid reactive substance (TBARS) assay kit (Cayman Chemical) according to manufacturer’s instructions. In brief, the stomach was homogenized with lysis buffer (150 mM NaCl, 1% Triton X-100, 0.5% sodium deoxycholate, 0.1% sodium dodecyl sulfate (SDS), 50 mM Tris, pH 8.0) and then the homogenates were centrifuged at 3500 rpm for 30 min. The supernatant (250 μL) was added to 10% trichloroacetic acid (250 μL), and the mixture was centrifuged at 3000 rpm for 15 min. After centrifugation, the supernatant (100 μL) was mixed with 0.67% thiobarbituric acid (100 μL) and was incubated at 100 °C for 15 min. Then, the absorbance was measured at 532 nm using the Synergy™ HTX Multi-Mode Microplate Reader (BioTek Instruments).

### 2.13. Measurement of Superoxide Dismutase (SOD) Activity

SOD activity was measured by SOD Assay Kit (Dojindo, Kumamoto, Japan) following to the manufacturer’s instructions. In brief, the stomach was homogenized with sucrose buffer (0.25 M sucrose, 10 mM Tris, 1 mM EDTA, pH 7.4) and the homogenate was centrifuged at 4 °C for 1 h at 12,000 rpm. The supernatant was diluted with diluent buffer and added to water soluble tetrazolium salt (WST) working solution. After 20 min incubation, the absorbance was analyzed at 450 nm using the Synergy™ HTX Multi-Mode Microplate Reader (BioTek Instruments).

### 2.14. Measurement of Intracellular Reactive Oxygen Species (ROS)

HMC-1 cells (3 × 10^6^) were seeded at 6-well plates and were pre-treated with α-humulene (1 mM) for 1 h and then followed by PMA (20 ng/mL) treatment for 4 h. Cells were washed with PBS and stained with CellROX (5 μM, a cell permeable fluorescent reagent for detecting oxidative stress, Life Technologies, Carlsbad, CA, USA) at 37 °C for 30 min. Following washing with PBS, the cells were fixed in 4% paraformaldehyde for 15 min and nuclei were counterstained with Hoechst 33342 (Life Technologies) to detect nuclei for 10 min. Images of cells were obtained with the Axiovert M200 microscope (Zeiss).

### 2.15. RNA Isolation and Real-Time Polymerase Chain Reaction (PCR)

HMC-1 cells (3 × 10^6^ in a 6-well plate) were cultured either without or with varying concentrations of α-humulene for 1 h and then followed by PMA (20 ng/mL) treatment for 4 h. Total RNAs were isolated from HMC-1 cells with TRI Reagent (Sigma) and reverse transcribed to cDNA using M-MLV reverse transcriptase kit (Promega). For gastric tissues, rat stomach was dissected and homogenized with TRI Reagent, and transcribed to cDNA by reverse-transcription. Real-time PCR was performed on a Rotor-Gene Q cycler (Qiagen, Redwood City, CA, USA) using the Rotor Gene SYBR Green RT-PCR kit (Qiagen) following the manufacturer’s instructions. The PCR primers were purchased from Bioneer (Daejeon, Korea) and are as follows: human β-actin (forward, 5′-agagctacgcgctgcctgac-3′; reverse, 5′-actcacctcttcagaacgaattg-3′); human interleukin-1β (IL-1β, forward, 5′-atgatggcttattacagtggcaa-3′; reverse, 5′-atgatggcttattacagtggcaa-3′); human TNF-α (forward, 5′-cctctctctaatcagccctct-3′; reverse, 5′-gaggacctgggagtagatgag-3′); human IL-6 (forward, 5′-gtgttgcctgctgccttc-3′; reverse, 5′-agtgcctcttgctgctttc-3′); rat β-actin (forward, 5′-agagctacgagctgcctgac-3′; reverse, 5′-agcactgtgttggcgtacag-3′); rat trefoil factor 1 (TFF1, forward, 5′-agtgcaaggaaaagggttgc-3′; reverse, 5′-tctcaatgaccagaggtcgg-3′); rat TFF2 (forwward, 5′-cccacttccaaaccaagcgtcg-3′; reverse, 5′-cagcagtgcccttcagtagt-3′); rat TFF3 (forward, 5′-caaatgtgccctggtgcttc-3′; reverse, 5′-cccagctcctatattggccg-3′); rat mucin 5AC (MUC5AC, forward, 5′-acaacagacagacgtgacgg-3; reverse, 5′-gtcatctggacagaagcagccctc-3′); rat MUC6 (forward, 5′-agggggagaaacagcctaca-3′; reverse, 5′-aacacattgcaccaatcccg-3′); rat polymeric immunoglobulin receptor (pIgR, forward, 5′-agtcgctctgttgtgaaggg-3′; reverse, 5′-acaccagtacttgaggtgc-3′).

### 2.16. Western Blotting

HMC-1 cells (5 × 10^7^ in a 100 mm dish) were cultured either without or with varying concentrations of α-humulene for 1 h and then followed by PMA (20 ng/mL) treatment for 4 h. Following the treatment, the cells were homogenized with buffer A (10 mM HEPES, 1.5 mM MgCl_2_, 10 mM KCl and 0.5 mM DTT) and the homogenates were centrifuged at 2500 rpm for 5 min at 4 °C. The supernatant was collected to following centrifugation at 13,000 rpm for 20 min and labeled the cytosolic fraction. The pellets were resuspended in buffer C (20 mM HEPES, 1.5 mM MgCl_2_, 0.5 mM DTT, 20% glycerol, 0.2 mM EDTA and 0.42 mM NaCl) and centrifuged at 13,000 rpm for 30 min at 4 °C. The supernatant was labeled the nuclear fraction. Nuclear and cytoplasmic proteins were resolved using 10% sodium dodecyl sulfate-polyacrylamide gel electrophoresis and transferred to nitrocellulose membranes. All membranes were incubated primary antibodies against inhibitor of nuclear factor kappa B (I-κB), nuclear factor kappa-light-chain-enhancer of activated B cells (NF-κB), glyceraldehyde-3-phosphate dehydrogenase (GAPDH), and proliferating cell nuclear antigen (PCNA) in blocking solution for 18 h at 4 °C. After washing with PBS, the membranes were incubated with the secondary antibodies at room temperature for 1 h. The protein bands were detected by chemiluminescence (FUSION-SL4, Vilber, Collégien, France).

### 2.17. NF-κB Luciferase Reporter Assay

HMC-1 cells (2.0 × 10^6^) were transfected with 0.1 μg of the pGL2-NF-κB-Firefly-Luciferase reporter plasmid (Promega) and 0.01 μg of the Renilla-Luciferase reporter plasmid (pTK-Renilla, Promega) using a FuGENE 6 transfection reagent (Roche, IN, USA). Following transfection for 48 h, the cells were pre-treated in the absence or presence of α-humulene for 4 h and then incubated with PMA (20 ng/mL) for 1 h. Then, the luciferase activity was measured by Dual-Luciferase Reporter Assay Kit (Promega) according to the manufacturer’s instruction.

### 2.18. Immunofluorescent Staining

HMC-1 cells (2 × 10^5^ in a 24-well plate) were cultured either without or with varying concentrations of α-humulene for 1 h and then followed by PMA (20 ng/mL) treatment for 4 h. Following the treatment, the cells were washed with PBS, fixed for 20 min using 4% paraformaldehyde, and permeabilized with 0.3% Triton X-100 for 15 min. Then, cells were incubated with anti-p65 antibody (1:100, Cell Signaling Technologies) in a blocking solution for 16 h at 4 °C. After washing with PBS, cells were incubated with Alexa 488-conjugated IgG (1:1000, Life Technologies) for 50 min. Nuclei were counter-stained using Hoechst 33342 (Life Technologies). Fluorescent images were obtained with an Axiovert M200 microscope (Zeiss).

### 2.19. Statistical Analysis

Data are presented as the means ± standard deviation. Statistical significance was determined using a two-sided Student’s *t*-test or one-way ANOVA. *p* values < 0.05 were considered significant. All significant differences were based on comparisons with the control group or HCl-ethanol-treated (positive control) group and are indicated in the figures with plus signs (+ *p* < 0.05, ++ *p* < 0.01, and +++ *p* < 0.001) or asterisks (* *p* < 0.05, ** *p* < 0.01, and *** *p* < 0.001), respectively.

## 3. Results

### 3.1. α-Humulene Attenuates Mucosal Lesions in an HCl/Ethanol-Induced Gastritis Model

**Figure 1:**
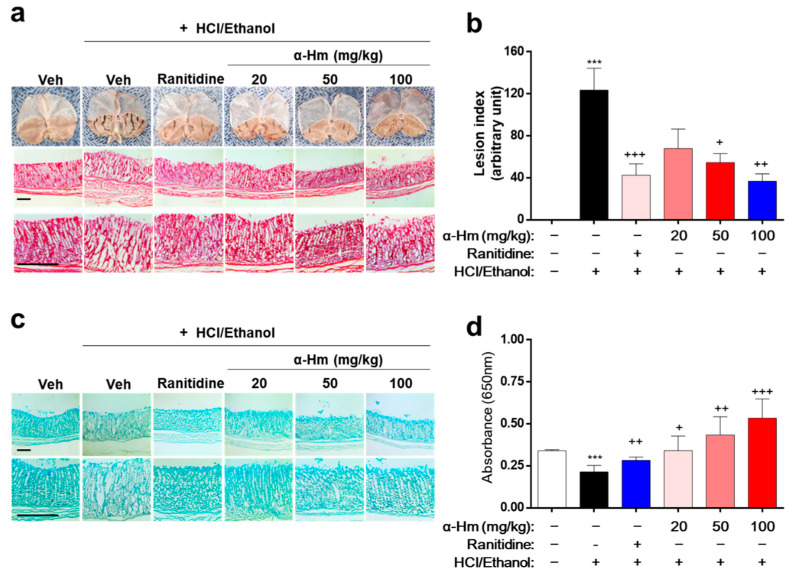
α-Humulene attenuates the mucosal lesions in HCl/ethanol-induced gastritis model. Either vehicle (Veh, Saline), α-humulene (α-Hm, 20, 50, or 100 mg/kg) or 50 mg/kg ranitidine was orally administered before intragastric HCl/ethanol administration in rats. (**a**) Representative images of acute gastric damage and histological investigation by hematoxylin-eosin (H-E) staining of stomach sections. Scale bars are 0.5 mm. (**b**) Graphs showing the lesion index (arbitrary unit). (**c**) Representative pictures of Alcian Blue staining of mucus glands. (**d**) Quantification of Alcian Blue by measuring absorbance at 650 nm after solubilizing the stained tissues. + *p* < 0.05, ++ *p* < 0.01, +++ *p* < 0.001, *** *p* < 0.001.

To investigate whether α-humulene has protective efficacy against gastric mucosal damage in vivo, histological analysis was performed in an HCl/ethanol-treated rat model. Thirty minutes before the HCl/ethanol-induced lesions, α-humulene (20, 50, or 100 mg/kg) was administered orally. Ranitidine, a drug widely prescribed for peptic ulcers, was used as a positive control. As shown in Figure 1a, the gastric lesions, which appear like fingernail scratches, were observed in the HCl/ethanol groups but not in the vehicle-treated group. When the rats were pretreated with α-humulene, the gastric hemorrhagic lesions decreased in a dose-dependent manner. In particular, the group treated with 100 mg/kg of α-humulene showed anti-gastritic effects similar to that of ranitidine. With respect to gastric lesion inhibition (%), that of ranitidine and 100 mg/kg α-humulene was 65.65 ± 4.37% and 70.29 ± 10.19%, respectively (Figure 1b). Moreover, mucus production, which was greatly reduced by HCl/ethanol, was restored by α-humulene treatment in a dose-dependent manner (Figure 1c); this finding was corroborated by Alcain Blue quantification (Figure 1d). In addition, we further investigated whether α-humulene affects the gastric secretion in vivo by analyzing the volume of gastric acid secretion and titratable acidity in a pyloric ligation-induced gastritis model. Ranitidine, a well-known histamine H2-receptor antagonist, showed a notable decrease of the gastric secretion volume (5.67 ± 1.45). As shown in Table 1, α-humulene showed a significant reduction in gastric volume (2.41 ± 0.61) compared with the control group (6.60 ± 1.0), which received normal saline. Additionally, administration of α-humulene resulted in a significant reduction in titratable acidity by 50.95% when compared to control group. These data suggest that α-humulene prevented HCl/ethanol-induced gastric injury in a dose-dependent manner.

### 3.2. α-Humulene Increases mRNA Expression Levels of Mucus-Stabilizing Factors in HCl-Ethanol-Injured Stomach Tissues

**Figure 2:**
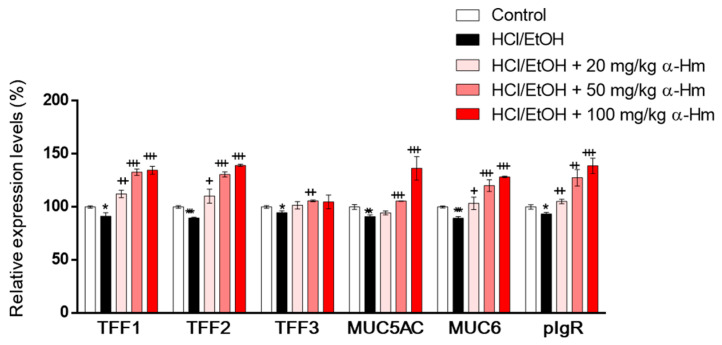
α-Humulene increases the mRNA expression levels of mucus-stabilizing factors in HCl-ethanol-injured stomach tissues. The mRNA expression levels of mucus-stabilizing genes mucin 5ac (Muc5ac), Muc6, trefoil factor 1 (Tff1), Tff2, Tff3 and polymeric immunoglobulin receptor (pIgR) are shown. α-Hm: α-Humulene. + *p* < 0.05, ++ *p* < 0.01, +++ *p* < 0.001, * *p* < 0.05, ** *p* < 0.01, *** *p* < 0.001.

To examine whether α-humulene increases the expression of mucus-stabilizing genes such as secretory mucins (Muc5ac and Muc6), secretory peptides (Tff1, Tff2 and Tff3) and Pigr, RT-PCR was performed using gastric epithelium tissues. When injured by HCl/ethanol, the mRNA expression levels of the mucus-stabilizing genes, Muc5ac, Muc6, Tff1, Tff2, Tff3, and Pigr were decreased (Figure 2). However, α-humulene increased the expression levels of Muc5ac, Muc6, Tff1, Tff2, and Pigr, but not Tff3, in a dose-dependent manner. These data suggest that α-humulene can increase the levels of mucus-stabilizing factors dose-dependently in HCl/ethanol-induced gastritis model.

### 3.3. α-Humulene Decreases MDA Levels and Enhances of PGE_2_ Expression and SOD Activity

**Figure 3:**
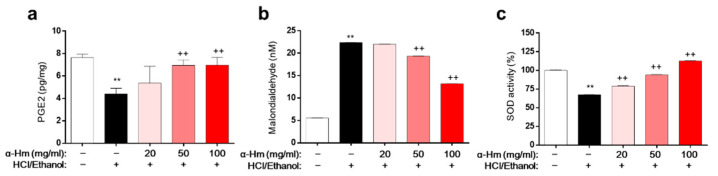
α-Humulene suppresses MDA formation and enhances PGE_2_ levels and SOD activity. (**a**) Prostaglandin E_2_ (PGE_2_) levels in stomach tissues. (**b**) Levels of malondialdehyde (MDA). (**c**) Relative superoxide dismutase (SOD) activity. α-Hm: α-Humulene. ++ *p* < 0.01, ** *p* < 0.01.

To assess the underlying molecular mechanism of α-humulene activity, biochemical evaluations of the levels of prostaglandin E_2_ (PGE_2_, mucus protective factor), superoxide dismutase (SOD, anti-oxidant enzyme), and malondialdehyde (MDA, lipid peroxidation marker) in HCl/ethanol-induced gastritis model were performed. Compared to those of the vehicle-treated group, the level of PGE_2_ and the activity of SOD were markedly lower and the level of MDA was dramatically higher in the gastric tissues of the HCl/ethanol-treated group (Figure 3a–c). However, α-humulene prevented this decrease in PGE_2_ level and SOD activity and increase in MDA levels. These data suggest that α-humulene decreases MDA levels and increases PGE_2_ level and SOD activity dose-dependently in HCl/ethanol-induced gastritis condition.

### 3.4. α-Humulene Has a Weak Acid-Neutralizing Capacity

To examine how α-humulene attenuates HCl/ethanol-induced gastric injury, the neutralizing capacity of α-humulene against artificial gastric juice was evaluated. Calcium carbonate (CaCO_3_) is known to be effective in relieving pain in the treatment of gastritis and gastric ulcers and hence was used as a positive control. CaCO_3_ showed 71.71% neutralization capability, while that of α-humulene was 11.18% (Table 2). Therefore, the protective action of α-humulene against HCl/ethanol-induced gastric injury was not driven by direct neutralization.

### 3.5. α-Humulene Inhibits Histamine Release in HMC-1 Cells through Ca^2+^ and Cyclic Adenosine Monophosphate

**Figure 4:**
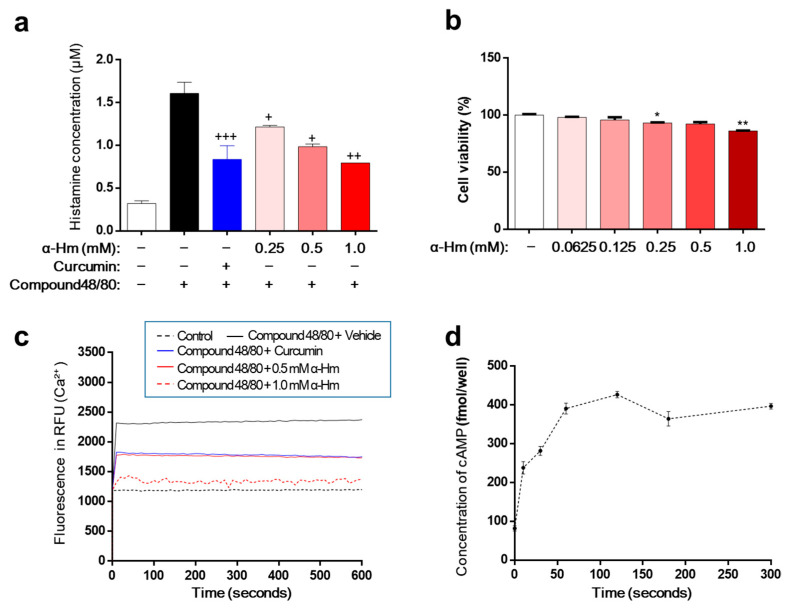
α-Humulene inhibits histamine release in HMC-1 cells. (**a**) Effect of α-humulene (α-Hm) on histamine release in compound 48/80-treated HMC-1 cells. HMC-1 cells were pre-incubated with α-humulene for 2 h and then stimulated with 5 μg/ml compound 48/80. Histamine levels in the conditioned medium were measured by spectrofluorimetry. Curcumin (50 μM) was used as a positive control. (**b**) Graphs showing cell viability using the MTS assay. (**c**) Effect of α-humulene on intracellular calcium influx in HMC-1 cells. (**d**) Effect of α-humulene on intracellular cyclic adenosine monophosphate (cAMP) levels in HMC-1 cells. + *p* < 0.05, ++ *p* < 0.01, +++ *p* < 0.001, * *p* < 0.05, ** *p* < 0.01.

Excessive secretion of histamine is the main cause of mucosal lesions with gastritis and gastric ulcers. Therefore, we investigated the effect of α-humulene on the secretion of histamine using human mast cell line-1 (HMC-1). When stimulated with compound 48/80, HMC-1 cells released histamine, and the concentration of histamine increased from 0.32 ± 0.01 μM to 1.61 ± 0.07 μM (Figure 4a). As a positive control, curcumin showed a strong inhibitory effect on histamine secretion. α-Humulene demonstrated a concentration-dependent reduction in histamine levels; especially, 1.0 mM α-humulene exhibited an inhibitory activity that was as strong as curcumin. Cell viability was examined to determine whether this inhibitory activity was due to cytotoxicity. α-Humulene showed little toxicity in the concentration range of 0.0625 to 1.0 mM (Figure 4b). Thus, the inhibitory activity of α-humulene on histamine secretion was not caused by cellular toxicity. Considering the main role of intracellular calcium (Ca^2+^) signaling in mast cell degranulation, we investigated whether α-humulene affects compound 48/80-induced Ca^2+^ mobilization in HMC-1 cells. Fura 2-acetoxymethyl ester (Fura/2AM, Invitrogen), a membrane-permeant derivative of the ratiometric calcium indicator, was used to measure intracellular Ca^2+^. When treated with compound 48/80, the level of intracellular Ca^2+^ was dramatically increased in HMC-1 cells (Figure 4c). α-Humulene showed a strong suppressive effect on Ca^2+^ mobilization in a concentration-dependent manner; in particular, 1.0 mM α-humulene decreased the level of intracellular Ca^2+^ to as low as that of the control group. It is also known that increasing intracellular cyclic adenosine monophosphate (cAMP) levels is related to inhibition of the release of mast cell mediators [30]. For example, curcumin decreases histamine release from mast cells by increasing intracellular cAMP levels. This prompted us to measure the changes in cAMP levels in the cells. Similar to curcumin, α-humulene was found to increase cAMP in a concentration-dependent fashion (Figure 4d). These results suggest that α-humulene prevents degranulation through inhibition of intracellular Ca^2+^ mobilization and elevation of intracellular cAMP levels.

### 3.6. α-Humulene Inhibits Inflammation-Related Factors in PMA-Stimulated HMC-1 Cells

**Figure 5:**
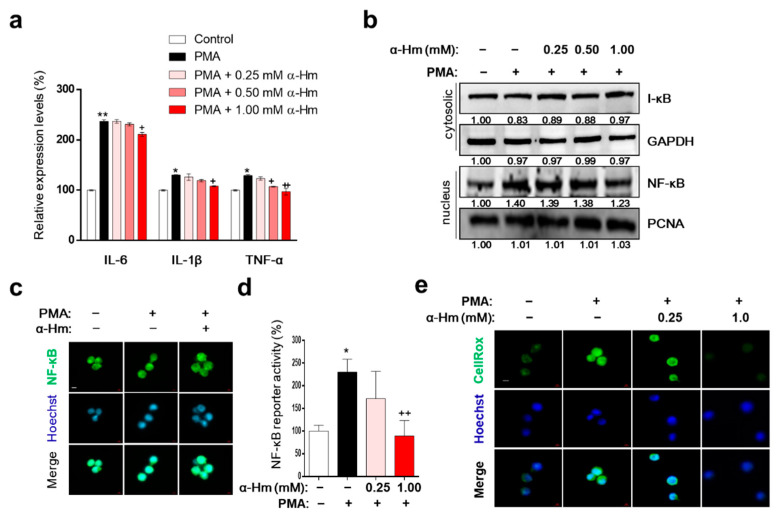
α-Humulene inhibits inflammation-related factors in PMA-stimulated HMC-1 cells. (**a**) mRNA expression levels of inflammatory cytokine genes interleukin (IL)-1β, tumor necrosis factor-α (TNF-α), and IL-6. HMC-1 cells were pretreated in the absence or presence of α-humulene (α-Hm) for 4 h and incubated with PMA (20 ng/ml) for 1 h. Glyceraldehyde 3-phosphate dehydrogenase (GAPDH) was used as the internal control. (**b**) Nuclear and cytosolic protein expression levels of nuclear factor kappa-light-chain-enhancer of activated B cells (NF-κB) and inhibitor of nuclear factor kappa B (I-κB). (**c**) Immunofluorescence staining of NF-κB in HMC-1 cells. Nuclei were stained with Hoechst 33342 (blue) and NF-κB was detected with anti-NF-κB antibody (green). All images are 40× magnification. (**d**) Transcriptional activity of NF-κB using luciferase reporter systems. (**e**) Effect of α-humulene on reactive oxygen species (ROS) production. The intensity of CellROX signal (a cell permeable fluorescent reagent for detecting oxidative stress, green) correlated with the amount of ROS. + *p* < 0.05, ++ *p* < 0.01, * *p* < 0.05, ** *p* < 0.01.

Interleukin (IL)-1β, tumor necrosis factor-α (TNF-α), and IL-6 are representative proinflammatory cytokines that promote the infiltration of leukocytes into the site of inflammation. As shown in Figure 5a, α-humulene suppressed the PMA-mediated increase in expression levels of inflammatory cytokine genes such as IL-6, IL-1B and TNF. Because nuclear factor kappa-light-chain-enhancer of activated B cells (NF-κB) upregulates the expression of IL-6 and IL-1B during acute gastric inflammation, we further examined whether α-humulene affects the NF-κB signaling pathway. When the cells were pretreated with PMA, NF-κB translocated to the nucleus; α-humulene attenuated the nuclear translocation of NF-κB in HMC-1 cells in a concentration-dependent manner (Figure 5b,c). In addition, α-humulene concentration-dependently decreased the transcriptional activation of NF-κB (Figure 5d). To examine whether α-humulene has radical-scavenging activity, CellROX^®^ reagent was used. α-Humulene suppressed PMA-induced ROS generation in a concentration-dependent manner in HMC-1 cells (Figure 5e). Taken together, α-humulene might inhibit NF-κB-mediated inflammatory responses in HMC-1 cells.

## 4. Discussion

Gastritis is an inflammation of the stomach, which results in chest pain, gastric hyperplasia, vomiting, and decreased appetite. The causes of gastritis vary and include *Helicobacter pylori* (*H. pylori*) infection, drinking alcohol, eating habits, drug abuse, and stress [31]. Various types of drugs are prescribed to treat gastritis including antacids, muscarinic receptor antagonists, and histamine receptor antagonists [32]. Ranitidine and cimetidine are the most widely used drugs, but they have side effects. Therefore, there is considerable demand for the development of new drugs to treat gastritis effectively with fewer side effects [33].

α-Humulene is a monocyclic sesquiterpene that is the main active constituent in many aromatic plants including *Humulus lupulus*. Various physiological activities of natural products containing α-humulene have been reported. For example, it has been reported that essential oils containing α-humulene not only have anti-allergic effects on carrageenan-induced foot edema models, but they also possess potent anti-inflammatory activity [34,35]. Moreover, essential oils containing α-humulene exhibit selective anti-tumorigenic activity [36,37]. The biological relevance of α-humulene itself has now started to be reported. For example, Chen et al. reported that α-humulene inhibits Akt (also known as protein kinase B) signaling and exerts an anti-cancer effect on hepatocellular carcinoma cells [38]. Kang et al. reported that α-humulene possessed antibacterial and antibiofilm efficacy against *Bacteroides fragilis* [39]. However, it is largely unknown whether α-humulene possesses beneficial activity against acute gastritis and how it affects the gastric mucosa. This prompted us to study the efficacy of α-humulene in protecting against HCl/ethanol-induced gastric mucosal damage and its underlying molecular mechanism.

There are various pathological mediators of gastritis and gastric ulcers, and histamine has a stimulating effect on gastric acid release [40]. Enterochromaffin-like cells secrete histamine, which stimulates parietal cells through the histamine 2 receptor (H2R) to secrete acid, resulting in a decrease in pH. Under stress conditions such as alcohol abuse, the level of histamine is elevated, which increases acid production, thus inducing gastritis. Therefore, we investigated the inhibitory effect of α-humulene on histamine release using HMC-1 cells. When stimulated with compound 48/80 or PMA, HMC-1 cells release histamine [41,42]. Here, we showed that α-humulene significantly decreased histamine secretion without cellular toxicity. Previous studies have shown that both intracellular calcium and cAMP act as important modulators during the degranulation of HMC-1 cells [43,44]. When mast cells are stimulated, calcium channels rapidly open at the membrane, and a large amount of calcium enters the cytoplasm [45]. Moreover, activated phospholipase C converts phosphatidylinositol 4,5-bisphosphate (PIP2) to inositol 1,4,5-trisphosphate (IP3), which binds to the IP3-gated calcium channel of the mast cell endoplasmic reticulum (ER). Then, a large amount of calcium stored in the ER is released into the cytoplasm. To investigate the underlying molecular mechanism of the antihistamine effect of α-humulene, the following experiment was conducted. First, we examined the changes in calcium influx using the fluorescent dye, Fluro-2/AM. Compared to compound 48/80-treated cells, α-humulene-treated cells showed lower levels of intracellular calcium. Next, we investigated the changes in cAMP, because intracellular cAMP increases to inhibit the release of mediators in mast cells [30]. Similar to curcumin (positive control), α-humulene increased intracellular cAMP levels, which resulted in the inhibition of histamine release. Taken together, α-humulene inhibits histamine secretion by regulating intracellular calcium and cAMP concentrations without any cytotoxicity.

Several studies have reported that oxidative damage plays an important role in HCl/ethanol-induced gastritis [46,47]. For example, free radicals are produced excessively, damage surrounding tissues, and exacerbate gastric injury. First, we tested the effect of α-humulene on ROS in HMC-1 cells in vitro and found that α-humulene normalized ROS levels. Second, PGE2 levels and SOD activity, which are representative mucus-protecting factors against oxidative stress-mediated gastritis, were investigated in gastric epithelial tissues [48]. In particular, PGE2 plays an important role in the maintenance of gastric mucosal integrity via several mechanisms, including the regulation of gastric mucosal blood flow, synthesis of mucus, and inhibition of gastric acid secretion. Interestingly, α-humulene increased both PGE2 levels and SOD activity in a concentration-dependent manner, resulting in a decrease in MDA, the most mutagenic product of lipid peroxidation [49]. Therefore, it was confirmed that α-humulene effectively inhibited superoxide radicals and lipid peroxidation through SOD and PGE2.

There is much evidence to support that the secretion of mucosal pro-inflammatory and immunoregulatory cytokines, including IL-1β, TNF-α, and IL-6, has a decisive effect on gastric inflammation [50]. It has been extensively reported that these cytokines are controlled by NF-κB. Previously, Ko et al. reported that Lindera erythrocarpa essential oil, with α-humulene as the main component, has anti-inflammatory effects in LPS-stimulated RAW264.7 cells through the inhibition of NF-κB [51]. Recently, Rogerio et al. reported that α-humulene relieved airway allergic inflammation through NF-κB [24]. In the present study, we revealed that α-humulene significantly decreased the levels of IL-1β and TNF-α and inhibited the activation of NF-κB. These results are consistent with the findings in prior studies. Based on the above results and previous reports, we concluded that α-humulene relieves gastric mucosal lesions through NF-κB inhibition.

In order to protect the stomach against various injuries such as stress and alcohol consumption, the presence of mucus is of paramount importance [52]. Interestingly, we found that mucus content increased significantly in the α-humulene-treated group, and we speculated that the anti-gastritic effect of α-humulene could be derived from mucus synthesis. To support this hypothesis, the gastric tissue was stained with Alcian Blue, which specifically binds to mucus [53]. As a result, we found that the amount of mucosa was decreased in the HCl/ethanol-administered group, and the decreased mucus was recovered by treatment with α-humulene. The gastric mucosa contains secretory mucins such as MUC5AC and MUC6, as well as other components that are secreted and stabilize the mucosal layer [54,55]. For example, the secretory peptides, TFF1 and TFF2, are secreted to protect the gastric mucus barrier [56]. In this study, we showed that the decreases in Tff1, Tff2, Muc5ac and Muc6 expression levels by HCl/ethanol were attenuated in a dose-dependent manner by α-humulene treatment. Therefore, the molecular mechanism of the anti-gastritic effect of α-humulene against HCl/ethanol seems to involve an augmentation in the gastric mucosal system by increasing the expression of gastric mucus. Further investigation should provide additional biochemical data to elucidate how α-humulene enhances the mucins including MUC5AC and MUC6.

Sesquiterpen is a type of terpene composed of three isoprene units and undergoes biochemical modifications (rearrangement or oxidation) producing the related sesquiterpenoids such as α-humulene and β-caryophyllene. Monocyclic sesquiterpene α-humulene is usually found together with its bicyclic isomer, β-caryophyllene, as well as its metabolites as a mixture in many flowers and plants such as *Lycopus australis* and *Eugenia caryophyllata* [57]. α-Humulene possesses an 11-membered ring with three *trans*-double bonds and β-caryophyllene has both a 9-membered ring with a *trans*-double bond and a nonpolar dimethylcyclobutane ring. The cyclobutane moiety of β-caryophyllene allows hydrophobic interactions with several target molecules. The differences in these structures can be attributed to the differences in α-humulene and β-caryophyllene representing different pharmacological activity. Rogerio et al. reported that α-humulene notably decreased the recruitment of eosinophils to the bronchoalveolar lavage fluid (BALF) in mouse airways allergic inflammation, but β-caryophyllene did not exhibit inhibitory effect. Sotto et al. reported that β-caryophyllene has a high affinity for P-glycoprotein (P-gp) in the HepG2, but α-humulene has a relatively low affinity for P-gp, which is explained by its open cyclic structure lacking in nonpolar cyclobutane moiety [58]. Since P-gp causes the multi-drug resistance by enhancing the efflux of anti-cancer drugs, α-humulene lacking this hydrophobic cyclobutane could be more useful as an anticancer drug than β-caryophyllene. In addition, Fernandes et al. reported that α-humulene inhibited the release of both TNF-α and IL-1β in carrageenan-induced paw oedema, while β-caryophyllene reduced only that of TNF α [34]. Singh et al. identified the interaction between three hydrophobic residues (Glu23, Glu24, and Asp140) of TNF-α and α-humulene through computational analysis as a related mechanism [59]. Furthermore, they reported that zerumbone, a structural analog of α-humulene having exocyclic carbonyl group, shows stronger binding to TNF-α, forming four hydrophobic interactions (Pro20, Gln21, Glu23, and Phe144 of TNF-α). These biochemical mechanisms might be involved in the suppressive effect of α-humulene on TNF-α shown in Figure 5a. Based on these reports, we are going to compare the efficacy and mechanism of α-humulene and its derivatives against mucosal injury and these structure–activity relationship studies could provide a pharmacophore of sesquiterpene having mucus stabilizing activity.

## 5. Conclusions

In this study, we found that: (1) oral administration of α-humulene significantly relieved HCl/ethanol-induced gastric lesions in vivo, (2) α-humulene inhibited histamine release from mast cells through Ca^2+^ mobilization and cAMP, (3) α-humulene increased the levels of mucus-stabilizing factors such as PGE2 and SOD, (4) α-humulene suppressed NF-κB-mediated pro-inflammatory responses, and (5) α-humulene enhanced mucus production by increasing the mRNA expression of genes such as Muc5ac and Muc6. Taken together, these results suggest that α-humulene exhibits protective efficacy against HCl/ethanol-induced gastritis in various ways including histamine, NF-κB, and mucus regulation; hence α-humulene could be developed as a drug candidate for the treatment of alcohol-induced gastritis.

## Figures and Tables

**Table 1:** Effect of α-humulene on the changes of gastric secretion volume and titratable acidity in pyloric ligation-induced gastric ulcers.

Groups	Volume (mL)	Titratable Acidity (Meq/L)
Control	6.60 ± 1.01	91.72 ± 17.36
Ranitidine	5.67 ± 1.45	64.95 ± 13.23
α-Humulene	2.41 ± 0.61 ^a^	44.98 ± 8.89 ^b^

Values are mean ± standard deviation (*n* = 5). ^a^^,b^ Means with the different letters in the column are significantly different (*p* < 0.05) by Duncan’s multiple range test.

**Table 2:** Acid-neutralizing capacity of α-humulene.

Material	NaOH Consumtion Volume (μL)	Acid-Neutralizing Capacity (%)
Control	50.67 ± 0.94	−
CaCO_3_	14.33 ± 2.62 **	71.71
α-Humulene	45.00 ± 0.82 **	11.18

α-Humulene (α-Hm) was mixed with artificial gastric acid and then incubated at 37 °C for 1 h in a shaking incubator. The neutralizing capacity was measured using 0.1 N NaOH, and phenol red was used as the pH indicator. ** *p* < 0.01.

## Data Availability

Not applicable.
